# MiR-19 Family Impairs Adipogenesis by the Downregulation of the PPARγ Transcriptional Network

**DOI:** 10.3390/ijms232415792

**Published:** 2022-12-13

**Authors:** Paula Juiz-Valiña, Bárbara María Varela-Rodríguez, Elena Outeiriño-Blanco, María Jesús García-Brao, Enrique Mena, Fernando Cordido, Susana Sangiao-Alvarellos

**Affiliations:** 1 Endocrine, Nutritional and Metabolic Diseases Group, CICA—Centro Interdisciplinar de Química e Bioloxía, Universidade de A Coruña, As Carballeiras, s/n, Campus de Elviña, 15071 A Coruña, Spain; 2 Endocrine, Nutritional and Metabolic Diseases Group, Department of Physiotherapy, Medicine and Biomedical Sciences, Faculty of Physiotherapy, Universidade de A Coruña, Campus de Oza, 15006 A Coruña, Spain; 3 Instituto de Investigación Biomédica de A Coruña (INIBIC), Xubias de Arriba, 84, 15006 A Coruña, Spain; 4 Department of Endocrinology, Hospital Universitario A Coruña, 15006 A Coruña, Spain; 5 Department of General Surgery, Hospital Universitario A Coruña, 15006 A Coruña, Spain

**Keywords:** adipogenesis, miR-19 family, morbid obesity, adipose tissue, bariatric surgery, PPARγ

## Abstract

microRNAs (miRNAs) are a class of small endogenous RNA that play pivotal roles in both the differentiation and function of adipocytes during the development of obesity. Despite this, only a few miRNA families have been identified as key players in adipogenesis. Here, we show the relevance of the miR-19 family, miR-19a and miR-19b, in lipid accumulation and the expansion of the adipose tissue in obesity. We observed that miR-19s were upregulated in the abdominal subcutaneous adipose tissue (aSAT) of human patients with morbid obesity, whereas after bariatric surgery, their expression was reduced. In vitro experiments identified miR-19a and b as crucial actors in adipogenesis and lipid accumulation. Overall, our results suggest a novel role of the miR-19 family in the regulatory networks underlying adipogenesis and, therefore, adipose tissue dysfunction.

## 1. Introduction

Obesity has increased over the past three decades, and the epidemiological data indicate that it is continuing to grow [1]. Currently, it affects more than 2 billion people worldwide [2] and it is one of the major contributors to metabolic syndrome, insulin resistance, type 2 diabetes mellitus (T2D), hypertension, hyperlipidemia, atherosclerosis and cardiovascular diseases. For all these reasons, it is considered a public health problem [3].

The development of obesity implies an abnormal expansion of white adipose tissue, which can occur due to both an increase in the size of the existing adipocytes and an increase in their number through the differentiation of new adipocytes. Adipogenesis is a complex process by which undifferentiated cells, called preadipocytes, become adipocytes [4]. Since this process determines the number of adipocytes, it could be a possible therapeutic target for obesity, and its study has acquired relevance in recent years [5]. Adipogenesis is regulated by a transcriptional cascade. During the early stages of differentiation, the CCAAT/enhancer-binding proteins (C/EBP), C/EBPβ and C/EBPδ, increase their expression. Then, C/EBPβ/δ stimulate C/EBPα and peroxisome proliferator-activated receptor γ (PPARγ), key transcription factors in adipogenesis and, finally, PPARγ and C/EBPα promote the induction of several adipocyte-specific genes in the terminal stage of differentiation [4,6]. Increasingly, data connect the dysregulation of microRNAs (miRNAs) with adipogenesis and obesity [7,8,9].

MiRNAs are small non-coding RNAs that can inhibit the translation of mRNAs. Different studies have shown that miRNAs are involved in nearly all developmental and pathological processes in animals [10,11]. In adipose tissue, miRNAs impact on energy homeostasis, metabolism, adipogenesis and the development of obesity, among other processes [4,7,9]. Different studies have also implicated miRNAs in adipogenesis regulation. The expression pattern of miRNAs changes throughout the adipogenic process, regulating it both positively and negatively. Proadipogenic miRNAs include miR-143, miR-125b-5p, miR-30a, miR-30d, miR-204, miR-211, miR-124, miR-210 and miR-146, while miR-130, miR-93 and the miR-27 family are antiadipogenic miRNAs (reviewed by [4]).

The miR-19 family is a conserved miRNA species that includes three members: miR-19a, miR-19b-1 and miR-19b-2. MiR-19a and miR-19b-1 are encoded by the miR-17-92 cluster, and miR-19b-2 is encoded by the miR-106a-363 cluster [12,13,14]. Although miR-19b-1 and miR-19b-2 are transcribed from different genes, they have the same mature sequence, which is called miR-19b. While miR-19a and miR-19b share many targeted genes, they can also act on different targets [13]. The miR-17-92 gene cluster is a highly conserved cluster that is distributed in vertebrates and encodes six miRs (miR-17, miR-18a, miR-19a, miR-19b-1, miR-20a and miR-92a) [15,16]. This cluster contributes to important biological processes and plays a role in many human diseases. It has been established as oncogenic [16] and also participates in the regulation of lipid and glucose metabolism [13,17]. Wang et al. demonstrated that during mouse preadipocyte 3T3-L1 cell differentiation, the overexpression of miR-17-92 accelerates adipocyte differentiation and triglyceride accumulation after hormonal stimulation [18]. Recently, a dual luciferase reporter gene assay applied to HEK-293T cells verified that miR-19b can bind to PPARγ, inhibiting its expression [19], which would contradict the notion of a proadipogenic function of miR-19b.

In order to understand the functional relevance of the miR-19 family during adipocyte differentiation, we overexpressed both miR-19a and miR-19b in 3T3-L1 preadipocyte cells and performed hormonal induction. We found that both miR-19a and miR-19b inhibited adipogenesis, although the changes were much more pronounced for miR-19b. We also found that both miR-19a and miR-19b increased their expression at the same time as the PPARG mRNA expression levels decreased in the abdominal subcutaneous adipose tissue (aSAT) of patients with morbid obesity, trends that are reversed after weight loss induced by bariatric surgery (BS).

## 2. Results

### 2.1. MiR-19a and miR-19b Expression Levels Decrease during 3T3-L1 Adipocyte Differentiation

**Figure 1:**
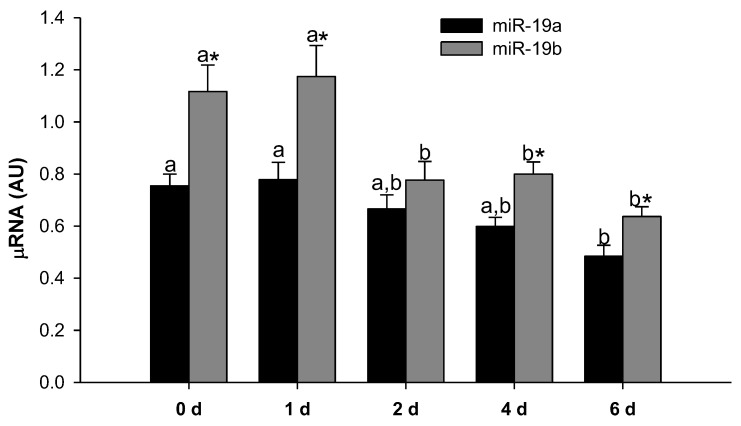
The miR-19 family expression decreases during adipogenesis of the 3T3-L1 cell line. Expression levels of miR-19a and miR-19b were analyzed in 3T3-L1 preadipocytes left untreated (0 d) and 1, 2, 4 and 6 days after inducing adipocyte differentiation. Data are represented as mean ± SEM in arbitrary units (AU) (n = 6–7). Different letters above the bars indicate statistical differences over time for each miRNA, and significance was determined by one-way ANOVA with post hoc Tukey’s test. * indicates differences between the two miR-19 family members for each time, and significance was determined by *t*-test analysis. d: day; SEM: standard error of the mean.

To evaluate the expression pattern of the miR-19 family during 3T3-L1 differentiation, we analyzed the miR-19a and miR-19b expression levels 1, 2, 4 and 6 days after inducing adipogenesis in the 3T3-L1 cell line, and we compared them with the undifferentiated cells. As shown in Figure 1, the expression level of miR-19a decreased in the late stages of cell differentiation, whereas the miR-19b values had already decreased in the intermediate stages of the differentiation process. It can also be seen that the expression values of miR-19b were higher than those of miR-19a, especially in the undifferentiated cells and in the earliest stages of differentiation.

### 2.2. Overexpression of the miR-19 Family Inhibits 3T3-L1 Cell Differentiation

**Figure 2:**
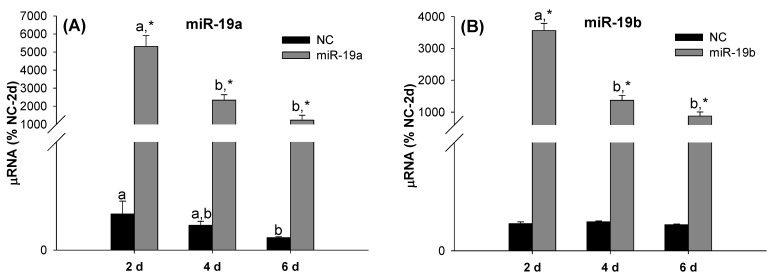
The miR-19a and miR-19b mimics efficiently increase the miR-19 family expression values. The miR-19a (**A**) and miR-19b (**B**) expression levels in 3T3-L1 cells transfected with miR-19a or miR-19b mimics at 2, 4 and 6 days after the induction of differentiation were estimated. Data are represented as mean ± SEM (n = 4) in arbitrary units, where 100% is equivalent to the control cells at day 2. Different letters above the bars indicate statistical differences over time for each treatment, and significance was determined by one-way ANOVA with post hoc Tukey’s test. * indicates differences by the transfection for each time, and significance was determined by *t*-test analysis. d: days, NC: mimic negative control, miR-19a: mimic for miR-19a, and miR-19b: mimic for miR-19b.

In order to further explore the relationship between the miR-19 family and 3T3-L1 cell differentiation, the 3T3-L1 cells were transfected with miR-19a and/or miR-19b mimics and the corresponding NC. As can be seen from Figure 2A,B, the miR-19a and miR-19b expression levels were significantly increased after transfection, and this increase was maintained throughout the experimental period (six days).

**Figure 3:**
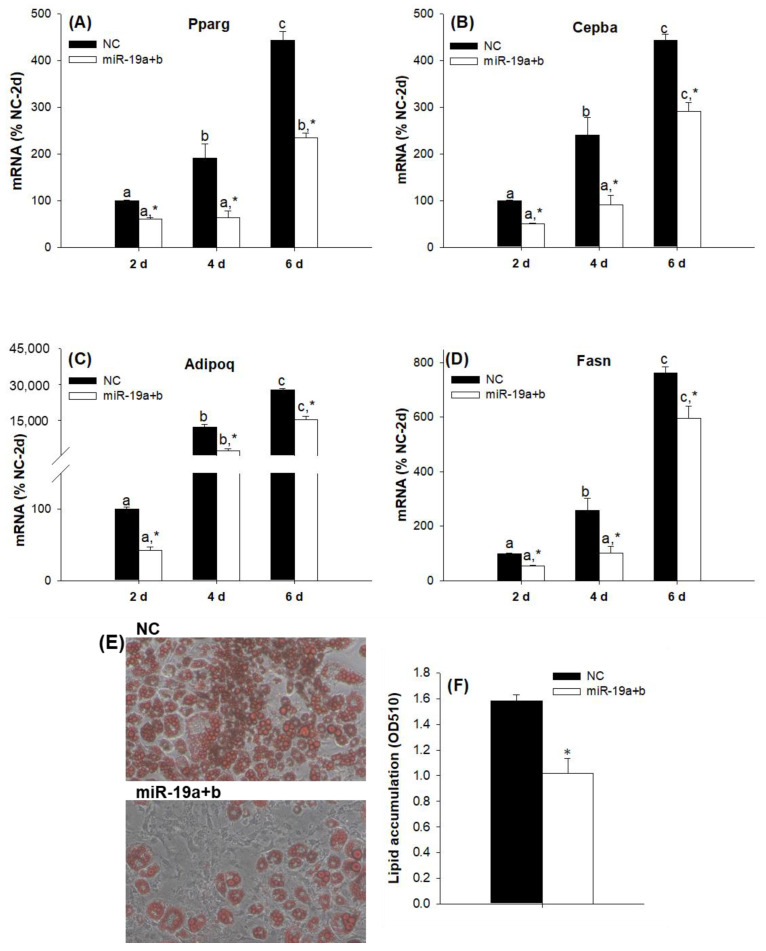
Transfection with the miR-19 family mimics inhibits adipogenesis in the 3T3-L1 cell line. The Pparg (**A**), Cebpa (**B**), Adipoq (**C**) and Fasn (**D**) mRNA expression levels were decreased during adipogenesis after transfection with the miR-19 family mimics. Data are represented as mean ± SEM (n = 8, two different experiments) in arbitrary units, where 100% is equivalent to the control cells at day 2. Different letters above the bars indicate statistical differences over time for each treatment (the Kruskal–Wallis test and post hoc Dunn’s test were performed). * indicates differences according to the transfection for each time (significance was determined by *t*-test or Mann–Whitney rank sum test analysis). The formation of lipid droplets was observed by light microscopy (Magnification: 10x) after the cells’ staining with Oil Red O (**E**), and the lipid accumulation at 6 days was determined by Oil Red O elution (**F**). Data are represented as mean ± SEM (n = 3). Significance was determined by *t*-test analysis, * *p* < 0.05 versus negative control mimic. d: days, NC: mimic negative control, miR-19a+b: mimics for miR-19a and miR-19b, simultaneously.

Figure 3 shows the effect of the simultaneous overexpression of miR-19a and miR-19b on the adipogenic differentiation marker genes and the ability of the transfected cells to accumulate lipids. The overexpression of miR-19a and miR19b significantly decreased the mRNA expression levels of adipogenic/lipogenic genes such as Pparg, Cebpa, Adipoq and Fasn (Figure 3A–D) during adipogenesis. The results of the Oil Red O staining were consistent with the above results (Figure 3E,F), since the overexpression of the miR-19 family significantly decreased the number of Oil-Red-O-stained cells (Figure 3E). The results of the Oil Red O extraction and quantification showed decreased lipid accumulation in the 3T3-L1 cells treated with the miR-19 family mimics with respect to the control cells (Figure 3F).

**Figure 4:**
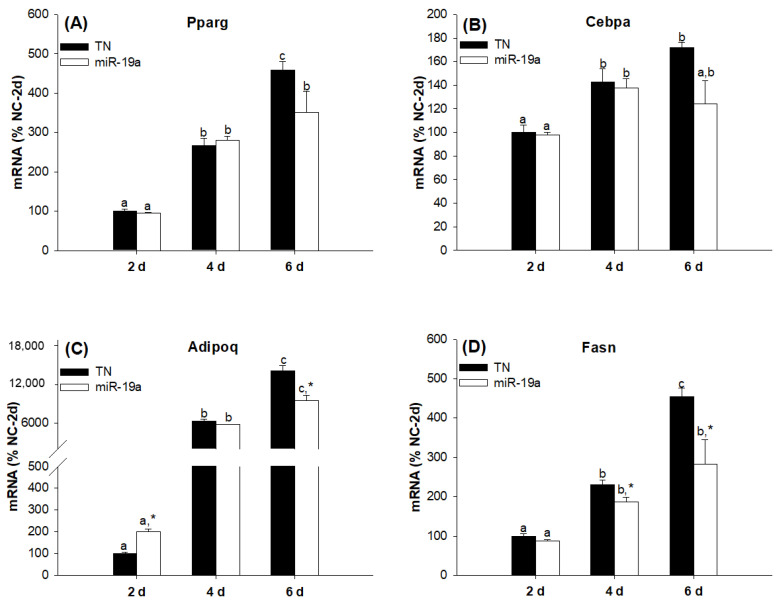
miR-19a modestly inhibits late adipogenesis in the 3T3-L1 cell line. The Pparg (**A**), Cebpa (**B**), Adipoq (**C**) and Fasn (**D**) mRNA expression levels were decreased during adipogenesis after transfection with the miR-19a mimic. Data are represented as mean ± SEM (n = 4) in arbitrary units, where 100% is equivalent to the control cells at day 2. Different letters above the bars indicate statistical differences over time for each treatment (significance was determined by the Kruskal–Wallis test with post hoc Dunn’s test or one-way ANOVA with post hoc Tukey’s test), and * indicates differences according to the transfection for each time (significance was determined by the *t*-test). d: days, NC: mimic negative control, miR-19a: miR-19a mimic.

**Figure 5:**
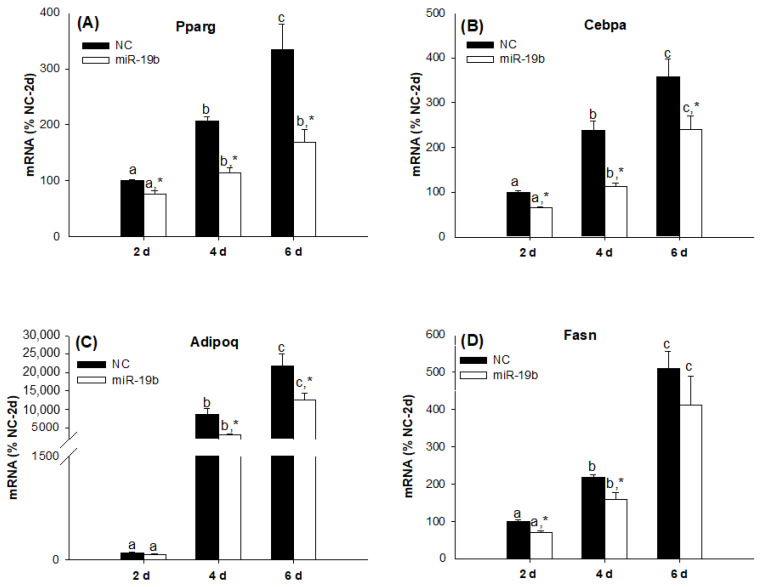
miR-19b inhibits adipogenesis in the 3T3-L1 cell line more markedly than mir-19a and in the earlier stages. The Pparg (**A**), Cebpa (**B**), Adipoq (**C**) and Fasn (**D**) mRNA expression levels were decreased during adipogenesis after transfection with the miR-19b mimic. Data are represented as mean ± SEM (n = 8, two different experiments) in arbitrary units, where 100% is equivalent to the control cells at day 2. Different letters above the bars indicate statistical differences over time for each treatment (significance was determined by the Kruskal–Wallis test with post hoc Dunn’s test or one-way ANOVA with post hoc Tukey’s test), and * indicates differences according to the transfection for each time (significance was determined by the *t*-test and Mann–Whitney rank sum test). d: days, NC: mimic negative control, miR-19a: miR-19a mimic.

Although, as many studies suggest, members of the same family of miRNAs may act in a functionally redundant manner [20], this is not necessarily the case. Therefore, our next step was to evaluate the individual effects of the overexpression of miR-19a and miR-19b on the differentiation process of 3T3-L1 cells. Despite the fact that the overexpression of both miRNAs regulated adipogenesis in the same direction, we found interesting differences between miR19a and miR19b. Whereas miR-19a overexpression modestly inhibited the adipogenesis of the 3T3-L1 cell line (Figure 4), miR-19b overexpression had a much more marked effect on this process (Figure 5). In fact, if we compare the respective differentiation time points, miR-19a overexpression did not alter the Pparg (Figure 4A) or Cebpa (Figure 4B) mRNA expression values and only decreased the Adipoq (Figure 4C) and Fasn (Figure 4D) mRNA values with respect to the negative control (NC) six days after inducing the differentiation process. However, when comparing the evolution of the expressions of Pparg (Figure 4A) and Cebpa (Figure 4B) over time for each group of cells, it was observed that in control cells, there was a more marked increase between days 4 and 6 compared to the group that were overexpressed. On the other hand, miR-19b overexpression inhibited the gene expression values of Pparg (Figure 5A), Cebpa (Figure 5B), Adipoq (Figure 5C) and Fasn (Figure 5D) in a more potent way than miR-19a and in the earlier differentiation stages.

### 2.3. Levels of miR19s in aSAT Are Modulated by Body Weight

**Figure 6:**
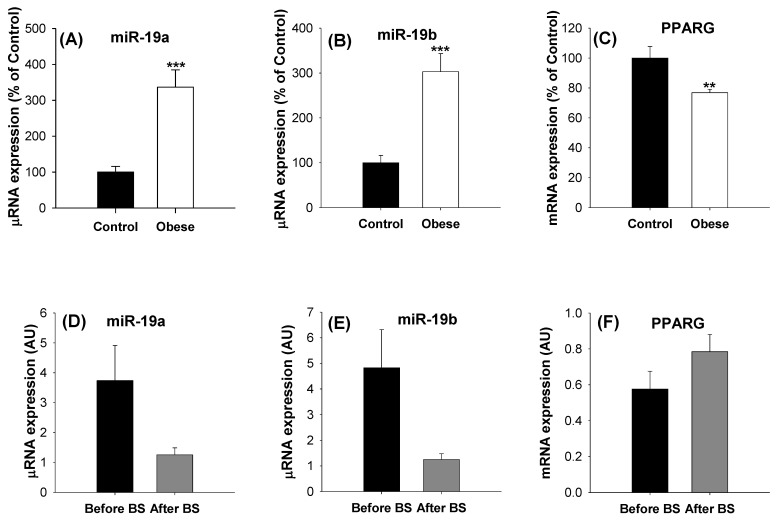
The miR-19 family expression levels are increased and the PPARG mRNA expression levels are decreased in the human aSAT of patients with morbid obesity. After body weight normalization induced by BS, these trends are reversed. The miR-19a (**A**), miR-19b (**B**) and PPARG (**C**) expression levels were analyzed in the aSAT of normal-weight control patients and morbidly obese patients. Data are represented as mean ± SEM (for miRNAs: Control n = 22 and Obese n = 42; for PPARG: Control n = 32 and Obese = 134) in arbitrary units, where 100% is equivalent to the control patients. Significance was determined by *t*-test analysis, ** *p* < 0.01 and *** *p* < 0.001 versus the control. After body weight normalization induced by BS, the miR-19a (**D**), miR-19b (**E**), and PPARG (**F**) expression levels were reanalyzed in six patients. Data are represented as mean ± SEM in arbitrary units. BS = bariatric surgery, AU = arbitrary units.

Taking into account the previous results, we decided to verify whether the miR-19 family and PPARG gene expression are deregulated in morbid obesity. To this end, we compared the expression levels of mir-19a, miR-19b and PPARG in aSAT tissue using qPCR applied to healthy patients with normal BMI (control group) versus morbidly obese patients. The characteristics of the subjects included in the studies and the comparison between the control and obese patients are summarized in Table 1. A sub-cohort of the patients used to analyze the PPARG gene expression was used for the miR-19a and miR-19b expression analyses. There were no statistically significant differences in age between the non-obese and obese patients, and the glucose levels, BMI and the percentage of diabetic patients were higher among the obese patients. The mir-19a and miR-19b expression levels tripled their values in the obese subjects compared to the control group (Figure 6A,B), and the PPARG expression decreased by almost 25% in the obese patients, as shown in Figure 6C. In order to verify whether the expression levels of these miRs are fully or partially restored after weight loss, the expression levels of miR-19a, miR-19b and PPARG were analyzed in six patients with morbid obesity and in the same patients after body weight normalization induced by bariatric surgery. The anthropometric parameters of these patients before and after bariatric surgery are shown in Table 2, and the results obtained for miR-19a, miR-19b and PPARG are presented in Figure 6D–F. It can be seen that after weight loss, the expression values of both members of the miR-19 family decreased considerably, while the PPARG gene expression level tended to increase; however, the trends did not reach statistical significance in any case.

## 3. Discussion

The present study establishes for the first time, to our knowledge, that the overexpression of the miR-19 family, especially miR-19b, inhibits the differentiation of 3T3-L1 cells, reducing the expression of the key transcription factors of adipogenesis, adipocyte-specific genes and lipid droplet accumulation. Moreover, the results presented in this work suggest that miR-19b overexpression impairs the intermediate stages of the adipogenic process through the inhibition of the Pparg transcriptional network, while miR-19a overexpression affects the later stages of the adipogenic process by altering mature adipocyte markers, such as Adipoq and Fasn. In addition, we also show that an increase in the gene expression of both members of the miR-19 family and a decrease in the expression of PPARG occur simultaneously in the aSAT of morbidly obese humans, and both patterns tend to reverse after weight loss induced by bariatric surgery.

The miR-19 family has been described as being involved in metabolism, tumorigenesis, inflammation, aging, tissue fibrosis and the regulation of the development of neurons, vessels and the heart [13,16,17,21,22,23,24,25,26]. The possible involvement of the miR-19 family in adipocyte function was first conceivable after the global stable transfection of 3T3-L1 cells with the miR-17-92 cluster showed a positive role in the clonal expansion phase of adipogenesis, accelerating adipocyte differentiation and increasing triglyceride accumulation [18]. This finding contrasts with the results obtained in our study. However, the former study was specifically focused on the early stages of adipogenesis, while we focused on a later stage, which could explain the observed differences. It must also be taken into account that although members of an miRNA cluster usually have the same targets or target different genes belonging to specific pathways [27], functional differences may also exist between the individual members. It should also be noted that this cluster is composed of miRs from four different families [28]. However, Wang et al. did not analyze the effects of each member (or family) of the cluster separately [18]. Therefore, functional differences between the different families, or even among the individual miRNAs that form this cluster, may explain the differences observed between these studies.

miRNAs are known for their ability to bind to the 3′ untranslated region (3′UTR) of specific mRNAs to induce mRNA degradation and translational repression [29]. One of the difficulties in identifying the miRNAs that mediate a biological process is that a single miRNA can directly regulate hundreds of mRNAs [30,31,32]. One of the quantitative assays most widely used to demonstrate the silencing of a putative target gene by a specific miRNA is the dual-luciferase reporter gene assay [33]. Wang et al., using this assay, verified that miR-19b can bind to PPARγ, inhibiting its expression [19], which is in line with the results observed in our study. We demonstrated that both miR-19a and miR-19b decrease their expression during 3T3-L1 cell adipogenesis (a process during which the Pparg expression levels increase), and when we forced their expressions simultaneously, we observed a decrease in lipid accumulation and in the gene expression of the key factors of the adipogenic process, such as Pparg, Cebpa and adipocyte-specific genes, such as Adipoq and Fasn. However, it is not possible to state definitively that the effect of the miR-19 family on PPARγ is direct. It cannot be ruled out that miR-19 has other targets that ultimately alter the expression levels of PPARγ.

In view of the results obtained in vitro, we sought to verify whether the expression levels of the miR-19 family and PPARG are deregulated in the adipose tissue in morbid obesity, and we found that they present opposite profiles. While the levels of miR-19a and miR-19b were markedly increased in the aSAT of obese patients, the PPARG gene expression was reduced. In addition, the normalization of body weight induced by bariatric surgery again exerted opposite changes in the miR-19 family and PPARG. While miR-19a and miR-19b experienced a slight decrease in their values, the PPARG values tended to increase, although in no case did the changes become statistically significant, probably due to the low number of patients. These results, again, support a negative relationship between the miR-19 family and PPARγ. Considering that PPARγ is the main regulator of adipogenesis and is required for adipocyte differentiation, insulin sensitivity, lipogenesis, and adipocyte survival [5,34], alterations in the miR-19 family could contribute to adipose tissue impairment.

In humans, there are two main adipose tissue depots, namely SAT and visceral adipose tissue (VAT). SAT is the best storage site for excess lipids, and its expansion is less harmful than that of VAT, preventing lipotoxicity in other tissues. However, SAT has a limited ability to expand [34,35,36]. Obese people with an increased adipogenesis capacity of SAT maintain a healthier metabolic state than those with impaired adipogenesis, as this leads to ectopic fat deposition in tissues such as the skeletal muscle or liver, contributing to insulin resistance and increasing the risk of type 2 diabetes. When the adipogenic process is inadequate, this leads to a hypertrophic expansion of cells, and people with large subcutaneous adipocytes have little capacity to differentiate preadipocytes into adipocytes, which is due to their impaired PPARγ activation [5,34,37].

Different studies have analyzed the adipose tissue mRNA expression of PPARG in obese patients, reporting conflicting and discrepant results. There are studies in which the PPARG gene expression in the adipose tissue (both subcutaneous and visceral) increases with obesity, while in others it decreases, and there are studies in which no changes were observed (reviewed by [38]). These inconsistent results may be due to differences in the BMI, age or sex of the patients studied. Another important point is the sample size. In many articles, the number of patients is very low, especially in the control group, where the number of patients is often less than 15. It should also be taken into account that many studies used a single reference gene, without providing data on its stability, to normalize the PPARG expression. Here, we analyzed 32 control patients and 134 obese patients of similar ages, a number much higher than that used in most of the studies, and we also used three reference genes simultaneously. Insulin sensitivity is another factor that can alter the PPARG expression values of adipose tissue. Some authors found that the PPARG mRNA expression increased [39,40] or did not change [41] in the SAT of normoglycemic obese patients compared to normoglycemic lean patients, but PPARG mRNA expression was significantly lower in prediabetic obese patients [41]. In this study, 40% of the patients used to analyze the gene expression levels of PPARG were diabetics. All patients diagnosed with diabetes are treated, mostly with metformin, and non-diabetic obese patients have higher glucose values than control patients. Therefore, it cannot be ruled out that they are patients in a prediabetic state. All of these variables can affect the results of PPARG expression. Overall, the data provided here suggest that the miR-19 family can downregulate PPARγ and impair adipogenesis.

Another suggestive point is the involvement of the miR-19 family in the regulation of inflammation [24,28,42,43]. It is known that obesity induces chronic low-grade inflammation of the adipose tissue, leading to a pro-inflammatory phenotype of the adipose-tissue-resident immune cells, especially the macrophages [44]. After weight loss, the number of macrophages and the expression of proinflammatory genes and proteins decrease in the adipose tissue [45,46]. Ortega et al. observed an increase in miR-19a and miR-19b in the supernatant of inflamed adipocytes, and miR-19a was also increased in the supernatant of inflamed macrophages. In line with our results, they also observed that, with weight loss induced by bariatric surgery, improved inflammation and the decreased expression of miR-19a and miR-19b in the SAT were detected [43]. Another study demonstrated that the miR-17~92 family miRNAs, consisting of three clusters, including miR-17-92 and miR-106a-363, which encode the different members of the miR-19 family, protect mice from obesity and control the balance between pro-inflammatory and anti-inflammatory phenotypes in the adipose tissue macrophages (ATMs). The myeloid-specific deletion of miR-17-92 family miRNAs drove the ATMs toward an inflammatory phenotype, leading to obesity and impaired glucose tolerance [28]. Given the above observations, the miR-19 family may participate in the regulatory networks underlying adipogenesis and inflammation and, therefore, adipose tissue dysfunction.

We must admit that this study has a number of limitations. Among them, we can highlight that in the human studies, we did not disaggregate the results by sex due, on the one hand, to the low number of women in the control group and, on the other hand, to the small number of patients who underwent abdominoplasty after bariatric surgery. Moreover, we did not take into account variables such as the heterogeneity of the obese patients with different comorbidities and treatments. Moreover, adipose tissue is not only composed of adipocytes but also preadipocytes, fibroblasts, endothelial cells and immune cells. Thus, it cannot be ruled out that the expression pattern of the miR-19 family is different in the different cells that comprise it. Therefore, in future studies using human adipose tissue, its different cellular components should be separated in order to carry out more detailed studies.

## 4. Materials and Methods

### 4.1. Cell Culture

The 3T3-L1 cells were obtained from the American Type Culture Collection (ATCC) and were cultured in Dulbecco’s modified Eagle’s medium (DMEM, Gibco, Spain) supplemented with 10% calf serum (HyClone, Cytiva, Spain) and antibiotics (100 units/mL penicillin G (P) and 100 µg/mL streptomycin (S), Gibco) at 37 °C in a humidified atmosphere containing 5% CO_2_.

### 4.2. 3T3-L1 Transfection

The 3T3-L1 preadipocytes were plated with 8 × 10^4^ cells per well on a 12-well plate and were transfected upon reaching 100% confluence with miR-19a-3p (cat. No. MC10649), miR-19b-3p (cat. No. MC10629) or a negative control (NC) mimic (cat. No. 4464058) at a final concentration of 20 nM (Applied Biosystems, Thermo Fisher Scientific, Inc., Madrid, Spain) in the presence of EndoFectin Max (GeneCopoeia, Rockville, MD, USA) at a final concentration of 3 µL EndoFectin/mL reduced serum media (OPTI-MEM, Gibco), following the manufacturer’s specifications, for 24 h. Subsequently, a 24 h recovery was carried out in a typical culture medium. The transfection efficiency was assessed by quantitative real-time polymerase chain reaction (qPCR).

### 4.3. 3T3-L1 Differentiation

To obtain fully differentiated adipocytes, the 3T3-L1 preadipocytes were induced to differentiation after their transfection recovery, i.e., 48 hours after reaching confluence, by adding a differentiation medium (0.5 mM 3-isobutyl-1-methylxanthine (IBMX, Sigma Aldrich)), 0.25 µM dexamethasone (Sigma Aldrich) and 1 μg/mL insulin (Actrapid) in Dulbecco’s modified Eagle’s medium/nutrient mixture F-12 (DMEM/F12, Gibco) supplemented with 10% fetal bovine serum (FBS, Gibco) and antibiotics (1% P/S). The IBMX and dexamethasone were removed after 2 days, but the insulin (1 μg/mL) was maintained for an additional 2 days. Thereafter, cells were grown in DMEM/F12 containing 10% FBS and antibiotics by replacing the media every 2 days.

### 4.4. Patients and Adipose Tissue Sample Collection

Abdominal SAT samples were obtained from three groups of patients: morbidly obese patients who underwent bariatric surgery (Roux-en-Y gastric bypass (RYGB) or sleeve gastrectomy (SG)), some of whom were had normalized their body weight (when the body mass index (BMI) was less than 30 kg/m^2^) and undergone abdominoplasty, and healthy non-obese patients who underwent abdominal hernia surgeries. All the samples were obtained during the surgeries, immediately placed on dry ice and then stored at −80 °C until further use. Written informed consent was obtained from all the patients, and the study was approved by the Research Ethics Committee of Galicia, Spain (reference 2014/135).

### 4.5. RNA Extraction and Rea-Time (RT) Quantitative (q)PCR

Total RNA from the cells and adipose tissue samples was isolated with TRIzol reagent (Invitrogen) following the manufacturer’s specifications. The RNA quality and its concentration were determined by agarose gel electrophoresis and spectrophotometry using a ND-100 Nanodrop 385 spectrophotometer (Thermo-Scientific, Madrid, Spain), respectively.

For the mRNA quantification of the cells, 1 μg of total RNA was retro-transcribed using M-MLV reverse transcriptase (Invitrogen, Madrid, Spain) and random primers (Invitrogen). Specific primer pairs and SYBR Green qPCR Master Mix (Roche, Madrid, Spain) were used for mRNAs quantification. The RT-qPCRs were performed using a Roche LightCycler 480 Real-Time PCR Detection System with the following program: one hold of 95 °C for 10 min, followed by 40 cycles of 15 s at 95 °C, 1 min at 60 °C and 5 s at 72 °C, followed by one hold of 72 °C for 10 min. The primer pairs used were as follows: Adipoq (NM_009605) forward 5′: TCCCAATGTACCCATTCGCT and reverse 5′: AACGGCCTTGTCCTTCTTGA; Cebpa (NM_001287514) forward 5′: ACTCGCTCCTTTTCCTACCG and reverse 5′: CCCCAACACCTAAGTCCCTC; fatty acid synthase (Fasn, NM_007988) forward 5′: GCCACCTCAGTCCTGCTATC and reverse 5′: GGTATAGACGACGGGCACAG; Pparg (NM_001127330) forward 5′: GGTGTGATCTTAACTGCCGGA and reverse 5′: ACCTGATGGCATTGTGAGACA; ribosomal protein S11 (Rps11, NM_013725) forward 5′: CATTCAGACGGAGCGTGCTTAC and reverse 5′: TGCATCTTCATCTTCGTCAC. For the data analysis, relative standard curves were constructed from serial dilutions of one reference sample of cDNA, and the input value of the target gene was normalized to S11.

For the mRNA quantification in the humans, 0.8 μg of total RNA per sample was retro-transcribed using SuperScript IV reverse transcriptase and random hexamers (Invitrogen). For the PCR, we used SYBR Green qPCR Master Mix (Roche). The primers used were: PPARG (NM_138712.5) forward 5′: GCTGACCAAAGCAAAGGCGA and reverse 5′: CAGCCCTGAAAGATGCGGATG; Importin 8 (IPO8; NM_006390.3) forward 5′: ACAATGTGTCTCCGTGCCAT and reverse 5′: AGCTTGCACTGCTCTGTGAT; hypoxanthine phosphoribosyltransferase 1 (HPRT1; NM_000194.3) forward 5′: TTGAGTTTGGAAACATCTGGAG and reverse 5′: GCCCAAAGGGAACTGATAGTC; glyceraldehyde-3-phosphate dehydrogenase (GAPDH; NM_002046) forward 5′: GAGTCCACTGGCGTCTTCAC and reverse 5′: GTTCACACCCATGACGAACA. For the data analysis, relative standard curves were constructed from serial dilutions of one reference sample of cDNA, and the input value of the target gene was standardized to the geometric mean of the three control genes, IPO8, HPRT1 and GAPDH, for each sample. PCR was initiated by one hold of 95 °C for 10 min, followed by 40 cycles of 15 s at 95 °C, 55 s at 60 °C and 5 s at 72 °C, followed by one hold of 72 °C for 10 min.

For the miRNA quantification (in both the humans and cells), 10 ng of total RNA was retro-transcribed using the TaqMan MicroRNA Reverse Transcription (RT) Kit (Applied Biosystems, Thermo Fisher Scientific, Inc.), according to the manufacturer’s instructions. MiRNA-specific stem-loop primers (miR-19a-3p, ref. 000395; miR-19b-3p, ref. 000396; and U6 small nuclear RNA (snRNA), ref 001973; Applied Biosystems, Thermo Fisher Scientific, Inc.) and TaqMan Universal Master Mix II, no UNG (Applied Biosystems, Thermo Fisher Scientific, Inc.) were used for the miRNA quantification according to the manufacturer’s protocol. RT-qPCR reactions were also performed using a Roche LightCycler 480 Real-Time PCR Detection System with the following program: 10 min pre-incubation at 95 °C, 40 cycles of 15 s denaturation at 95 °C and 60 s of elongation at 60 °C. For the quantitative miR-19a and miR-19b determination, U6 served as the internal reference.

### 4.6. Oil Red O Staining and Quantification

The cells were washed twice with PBS, fixed with 10% formalin for 1 hour and washed once with 60% isopropanol. Then, the cells were stained with Oil Red O solution (Sigma Aldrich) for 30 min. Following this, they were washed under tap water, and the lipid droplets were observed and photographed under a microscope (Nikon Eclipse TS 100, Nikon Europe, Amstelveen, The Netherlands) coupled with a digital XM Full HD Camera (XM Family, Samsung BWC-1602, Samsung, Madrid, Spain). Finally, the Oil Red O was eluted from the lipid droplets with 100% isopropanol and quantified using a microplate reader Sunrise™ (TECAN Group Ltd., Männedorf, Switzerland) by measuring the optical density (OD) at 510 nm.

### 4.7. Statistical Analyses

The data were analyzed using SigmaStat 3.1 software (Systat Software, Inc., Chicago, IL, USA), and the quantitative variables are presented as means ± standard error of the mean (SEM). Statistical significance was determined by *t*-test analysis (experiments with two groups) or one-way ANOVA with post hoc Tukey’s tests (experiments with more than two groups) if the data conformed to a normal distribution and the Mann–Whitney rank sum test (experiments with two groups) or Kruskal–Wallis test with post hoc Dunn’s test (experiments with more than two groups) if the data did not conform to a normal distribution. A value of *p* < 0.05 was considered significant. Qualitative variables were expressed as relative (%) frequencies, and significance was determined by the χ^2^ test.

## 5. Conclusions

Our findings suggest that miR-19 family may play a key regulatory role in adipose tissue adipogenesis through PPARγ inhibition. In addition, we showed that the aSAT of morbidly obese patients shows an increase in the gene expression of both miR-19a and miR-19b and a decrease in the expression of PPARγ. Since the maintenance of a healthy metabolic state depends on the correct adipogenesis in the SAT, an increase in the miR-19 family expression levels in obesity could contribute to the pathology of obesity, mediated by a decrease in the expression of PPARγ. However, more experiments are needed to confirm this hypothesis.

## Figures and Tables

**Table 1:** Characteristics of the patients included in the studies of the PPARG gene expression and miR-19 family expression. Quantitative variables are presented as the mean (±standard error of the mean), and qualitative variables are presented as %. Significance was determined by *t*-test analysis (quantitative variables) and the χ^2^ test (qualitative variables). * *p* < 0.05 versus the control.

	MiR-19 Family Study		PPARG Study	
	Control	Obese	Control	Obese
**Gender**	17 ♂ and 5 ♀	14 ♂ and 28 ♀	25 ♂ and 7 ♀	25 ♂ and 109 ♀
**Age (years)**	47.12 ± 2.26	43.49 ± 1.37	50.16 ± 2.29	46.47 ± 0.84
**BMI (kg/m^2^)**	24.05 ± 0.27	48.46 ± 1.23 *	24.75 ± 0.29	49.95 ± 0.74 *
**Glucose (mg/dL)**	86.64 ± 1.74	101.88 ± 4.95 *	88.81 ± 1.55	104.42 ± 2.42 *
**Diabetic patients (%)**	0%	40.48% *	0%	32.84% *

BMI: body mass index.

**Table 2:** Characteristics of obese patients before bariatric surgery and after body weight normalization induced by bariatric surgery. Values are expressed as the mean ± SEM. * *p* < 0.05 versus the control. Significance was determined by *t*-test analysis.

	Before Bariatric Surgery	After Bariatric Surgery
**Gender**	2 ♂ and 4 ♀	2 ♂ and 4 ♀
**Age (years)**	50.80 ± 5.00	53.13 ± 4.98
**BMI (kg/m^2^)**	48.52 ± 3.75	29.73 ± 0.98 *
**Glucose (mg/dL)**	96.83 ± 6.67	85.50 ± 2.58

BMI: body mass index.

## Data Availability

Not applicable.
